# Utility and Utilization of Patient-Reported Experience Measures for the Supplementary COVID-19 Protective Actions at the Ovidius Clinical Hospital in Romania

**DOI:** 10.3390/healthcare12030377

**Published:** 2024-02-01

**Authors:** Bogdan C. Pana, Ciprian Paul Radu, Florentina L. Furtunescu, Adrian Mociu, Nicolae Ciufu

**Affiliations:** 1 Department of Public Health, University of Medicine and Pharmacy Carol Davila, 050463 Bucharest, Romania; bogdan.pana@umfcd.ro (B.C.P.); florentina.furtunescu@umfcd.ro (F.L.F.); 2 Ovidius Clinical Hospital, 905900 Constanta, Romania; adrian.mociu@ovidius-ch.ro (A.M.); nicolae.ciufu@ovidius-ch.ro (N.C.)

**Keywords:** patient-reported measures, patient-reported experience measures (PREMs), patient perception, consumer self-reporting tools/instruments, hospital management, pandemic response, COVID-19 actions

## Abstract

Patient-reported experience measures (PREMs) provide assessments of patients’ subjective experiences and perceptions regarding their interactions with the healthcare system and its services. We present a cross-sectional study of the patient perception and evolution of COVID-19 cases performed at Ovidius Clinical Hospital in Romania during the COVID-19 pandemic. The study objective is to explore the utility and the utilization of PREMs in monitoring patient perceptions of the supplementary protective actions. During the pandemic, the hospital implemented early supplementary protective actions, like PCR and lung CT, to all surgically admitted patients in the hospital alongside government-recommended actions. At the same time, functional PREMs were used to evaluate patient perceptions regarding these supplementary actions. The research was carried out for 19 months between June 2020 and December 2021. The findings revealed that opinions about the severity of the COVID-19 pandemic, the personal risk of infection, and the perception of protective actions in the hospital were not correlated. Conclusions: The patients’ appreciation of the COVID-19 protective actions taken by the hospital is related more to the general perceptions induced by the number of cases presented in the mass media and less by perceptions of the gravity of the problem or the risk of infection. In a hospital, the primary mission of patient safety is essential, and it must be fulfilled even if the patients are not sure or fully convinced that this is for their benefit. For management decisions and monitoring, using PREMs can be essential in a situation when general evidence is not conclusive.

## 1. Introduction

Patient-reported experience measures (PREMs) are assessments of patients’ subjective experiences and perceptions relating to their interactions with the healthcare system and its services. Unlike patient-reported outcomes (PROMs), which focus on health status and treatment outcomes, PREMs are designed to capture patients’ views and feedback on the quality of care they receive and their overall experience with the health system—both from a macro and micro perspective [1]. They can be either relational, dealing with relations between patients and medical personnel, patients’ expectations, and preferences, or functional, focusing on basic expectations about technical issues related to delivering healthcare [2].

PREMs typically consist of questionnaires that ask patients to rate or describe various aspects of their healthcare experiences. Data are collected using different methods as follows: face-to-face interviews, self-completed questionnaires, or electronic data collection tools. The questionnaires are validated via different statistical tools that usually measure internal consistency, structural validity, and content validity [3].

The use of PREMs in hospital management can be extremely beneficial for calibrating management decisions but also has some operational difficulties. Challenges include the analysis methods, statistical tools, and specialists required who can provide results in a manner that is fast, reliable, and centered on patient needs, especially in a crisis situation when time constraints are high.

This aspect was also indicated by other authors who mentioned PREMs as relevant for triggering action at the micro level, and that there are not enough systematic evaluations of their resulting effects. Longitudinal research could provide more insight into patient experience enhancement strategies and the connections between PREM scores and the broader beneficial outcomes they are connected with [4].

The research context is that of the COVID-19 crisis. In Romania, the onset of the COVID-19 pandemic occurred at the end of February through the beginning of March 2020 (weeks 9–11/2020) [5], and the authorities followed World Health Organization (WHO) recommendations, implementing actions similar to those in other countries affected by COVID-19 [5]. Recommendations were issued by the Ministry of Health of Romania and were downloadable from the National Center for Transmittable Disease Control (CTDC) of the National Institute of Public Health (NIPH) [6].

Starting from 26 February 2020 [6], the recommendations specified the case definitions, triage schemes, and protective actions to be taken by medical institutions. The protective actions recommended at the national level had to be taken in all hospitals for the admission of patients as follows: a triage for all patients with a questionnaire (on symptoms, travel, contacts), non-contact temperature, PCR testing for positives at a high temperature, the admission in the green area for those patients who were negative, and the admission in the red zone for those patients who were positive.

Simultaneously, knowledge about the disease was advancing at a rapid pace, and different hypotheses, including both epidemiological and clinical evidence, had been built and circulated in the scientific environment in published or pre-print articles, including the low validity of non-contact temperature [7,8], the relevance of the chest CT in COVID-19 pneumonia evolution, and correlation with RT-PCR [9]. Some forms of evidence were taken into consideration late by the governmental agencies, but nevertheless, supplementary protective actions were permitted to be implemented if health facilities had the necessary resources [10].

The decision taken by OCH management team in March 2020 was to implement all possible protective actions, including supplementary protective actions (PCR and CT scan to all admitted patients). At the same time, the decision to monitor the impact of these decisions on patients’ perceptions using a questionnaire was taken.

The protective actions had both advantages and disadvantages at the population level. This was important to consider when making decisions about public health policies, but also for the decisions made in a hospital that relied on its brand and reputation for success. At the same time, there was uncertainty and a lot of debate about the outcomes of the protective actions forced into place at health facilities, the term used by the general public being “too many actions”.

Now, in 2023, we know that patients’ opinions about protective actions against COVID-19 in hospitals can vary greatly depending on factors such as individual beliefs, experiences, and concerns [11] but, at the time, all these actions were new and scary (as it is in all crisis situations).

A cross-sectional study is presented in this article with the purpose of sharing the experience of using PREMs by the management team of Ovidius Clinical Hospital (OCH), from Constanta county, Romania, in a crisis situation during the COVID-19 pandemic. This research was carried out between June 2020 and December 2021 for 19 months.

## 2. Materials and Methods

Objective: To explore the utility and the utilization of PREMs in monitoring patient perceptions of the supplementary protective actions taken by the hospital during the COVID-19 pandemic.

Design: A 19-month cross-sectional series of PREMs and COVID-19 incidence were studied from June 2020 to December 2021.

Study population: The study was conducted on 1465 questionnaires anonymously collected from patients admitted to the hospital in the period of June 2020–December 2021. The sample calculated for 95% confidence, 80% power, and +/− 3% error for an estimated 3600 admissions in 19 months of activity was 824. We decided to oversample at 1465 questionnaires, representing 40% of all admitted patients in the 19 months of the study.

Data collection: Data were collected using a questionnaire with 5 questions. The first question, Q1, was about the perception and magnitude of the COVID-19 problem; the second question, Q2, was related to the fear of COVID-19 infection; and the third question, Q3, concerned the amount of protective actions used in the hospital. For all three questions, a Likert scale with 5 points was used. Question four (Q4) was an open question about what supplementary protective actions should be used, and question five (Q5) was an open question about which actions should be dropped. The questionnaire used is presented in Appendix A.

The questionnaire was developed in the month of March 2020 by the hospital management team, including the quality department, research and development director, medical director, operational manager, and quality officer. The objective was to measure patient perceptions of the protective and overprotective COVID-19 actions, together with the opinions and fears related to the COVID-19 pandemic. We wanted also to assess if the three items of opinion, fear, and perceptions of protective actions are part of the same logical relationships, and whether the construct has an internal validity. The COSMIN Study Design checklist for patient-reported outcome measurement instruments was used as much as possible in the design and test of this PREM amidst the complex COVID-19 situation at the time [12].

Piloting of the first draft of the questionnaire was performed on 20 patients, half of whom were admitted, the other half being in outpatient treatment. They were asked to self-complete the printed questionnaire forms under the assistance of the hospital quality officer. After pilot testing, the wording and phrases were adjusted to assure better understanding and responsiveness. One of the frequent patient feedback items was that there is an exaggerate fear about COVID-19 by the health personnel and hospital and the protective actions are excessive. Stated plainly, the perception was that the hospital is taking “too many actions”. Therefore, the item “too many actions” was introduced as the status above “many” in question no.3 (Q3)—What do you think of the actions taken by OCH for protection against COVID-19: *Too many actions □ Many □ Enough □ Little □ No opinion □.* See Appendix A.

For 19 consecutive months, the questionnaires were self-completed by inpatients and outpatients and collected in a transparent closed feedback box. The quality department collected all questionnaires once per month and introduced data in an Excel database. The data analysis was performed by the research and development director, and presentations and evaluation were made by the hospital management team.

For operational decisions, during the 19-month period, the questionnaires for the previous month were analyzed during the first 5 working days of each month. The analysis was performed using MS Excel.

The measurements are the response rate for each option of a question in relation to the number of respondents to that question.

For the open-ended questions, Q4 and Q5, findings were classifying in 6 categories for each question. For Question 4, *What other actions to prevent the spread of COVID-19 do you think OCH could adopt? (If applicable)*, the categories were as follows: *1. None, 2. Operational related, 3. Personnel related, 4. Informational related*, and *5. System related*. All the other reported opinions were assigned into the category no. *6. Varia*. For details, see Table 1.

For Question 5, *In your opinion, which of the actions already adopted to prevent the spread of COVID-19 could OCH abandon? (If applicable)*, the categories were as follows: *1. None, 2. Masks, 3. CT scan, 4. PCR*, and *5. Triage*. All the other reported opinions were assigned into the category no. *6. Varia*. See Table 2.

The main variable that was followed by the management for operational monitoring and decisions was “*too many actions*” from the question Q3, this being identified by the management as the key answer reflecting opposition to protective actions.

For each month from June 2020 to December 2021, COVID-19 incidence at the national level in Romania was also collected. Data were downloaded from a public database with COVID-19 data [13] and converted from a weekly to monthly no. of cases. We used national data rather than local since these data were reported in national media outlets, which were the main drivers of opinion.

The evolution in the number of admissions in the hospital in the period 2017–2021 was extracted from the hospital database.

Data analysis on trends was performed in MS Excel 14.62 and SPSS 10. Cronbach’s alpha for internal validity, ratio response rates, and Pearson correlation coefficient (r^2^) for different comparison were calculated.

## 3. Results

We performed a validity check of the questionnaire after the first month of its reception. Cronbach’s alpha was calculated for the construct of Q1 and Q2 together at a value of 0.644. For the construct of Q1, Q2, and Q3 together, the value was 0.445; for Q1 and Q3, the value was 0.209; and for Q2 and Q3, the value was −0.132. Reverse coding for Q3 and recalculation of the Cronbach’s alpha for the same constructs produced the following results: for Q1, Q2, and Q3, the value was 0.451; for Q1 and Q3, the value was 0.330; and for Q2 and Q3, the value was −0.710. Based on these, we concluded that the opinions regarding the severity of the COVID-19 pandemic, personal risk of infection, and protective actions taken in the hospital went in different directions. Therefore, each of the questionnaire’s items was measured as the rate of total question responses and followed over time. From a managerial perspective, the most relevant findings were those of Q3—“too many actions”—and details from the answers collected from the questions Q4 and Q5. For the study objective, the data analysis of trends was performed for each year, 2020 and 2021, taking into consideration that, in January 2021, vaccination began, changing the general perception of the COVID-19 pandemic problem.

**Figure 1:**
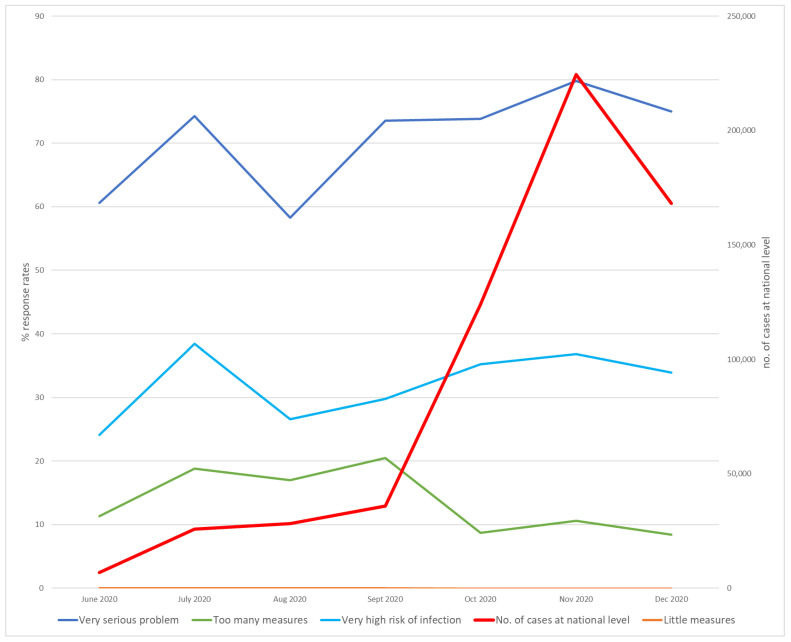
Evolution of the number of COVID-19 cases, infection risk perception, and the perception of protective actions in 2020.

In 2020, the proportion of respondents that considered the pandemic to be a problem was high (Figure 1). Between 60% and 74% of respondents rated it as a “very serious problem“ in the period of June–July 2020 when a high number of cases were reported, decreasing to 58% in August 2020 when the number of cases did not increase so much at a national level, and surging rapidly to 78% in November and 75% in December 2020 when the number of cases increased at a national level.

**Figure 2:**
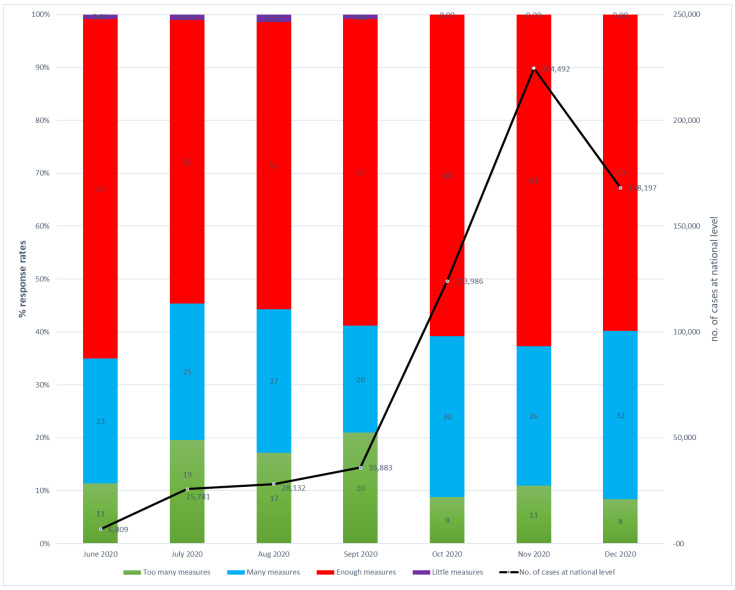
Opinions about the protective actions at OCH and the no. of cases in Romania in the period of June–December 2020.

Of the protective action items, “too many actions” constantly increased from 11% in June to 20% in September 2020 (Figure 2). This showed that patients started to become overloaded by the pandemic, and beliefs regarding the pros and cons of protective actions were fluid, raising the question if the supplementary protective actions should be kept in place or not. But in October 2020, the number of cases at a national level increased dramatically, and the “too many actions” responses dropped from 20% to 8%. The management’s decision was that supplementary protective actions should be kept in place at the OCH.

Correlation analysis provides some of the answers to these evolutions. The proportion of individuals who perceived the pandemic as a “very serious problem” correlated significantly with the “very high risk of infection” responses (r^2^ = 0.864, *p* = 0.012) and, in a smaller measure, with the number of cases, with a *p*-value close to the threshold (r^2^ = 0.707, *p* = 0.076). This confirms the logical construction that more cases = serious problems = high risk.

The item “too many actions” had an inversely proportionate correlation with the number of cases (r^2^ = 0.665, *p* = 0.013), and was not correlated with a “very serious problem” (r^2^ = 0.189, *p* = 0.685) or “very high risk of infection” (r^2^ = 0.137, *p* = 0.769).

Therefore, these observations reveal that when patients see the problem as being more serious, they positively appreciate supplementary protective actions, and the ratio of “too many actions” decreases.

Patients’ answers to the open-ended questions (Q4 and Q5) were less. On average, only 19% and 17% of the respondents in 2020 also answered the questions Q4 and Q5. For Q4, most of the answers were to re-confirm the actions taken. The category of “None” had the biggest proportion, with 68%. The other suggestions were regarding the categories of Operational with 15%, Varia with 7%, Personnel with 5%, System with 3%, and Information with 2%.

Similarly, on average, 82% of the Q5 respondents consider that none of the actions taken should be dropped. From the affirmative recommendations, they were mainly to drop masks with 8%, drop CT scan with 4%, drop tests with 3%, and Varia with 3%.

From a hospital management perspective, the supplementary actions implemented in 2020 were a successful strategy; actions were accepted by the patients, and the number of admissions increased to 9% compared with the previous year, 2019 (Table 3).

**Figure 3:**
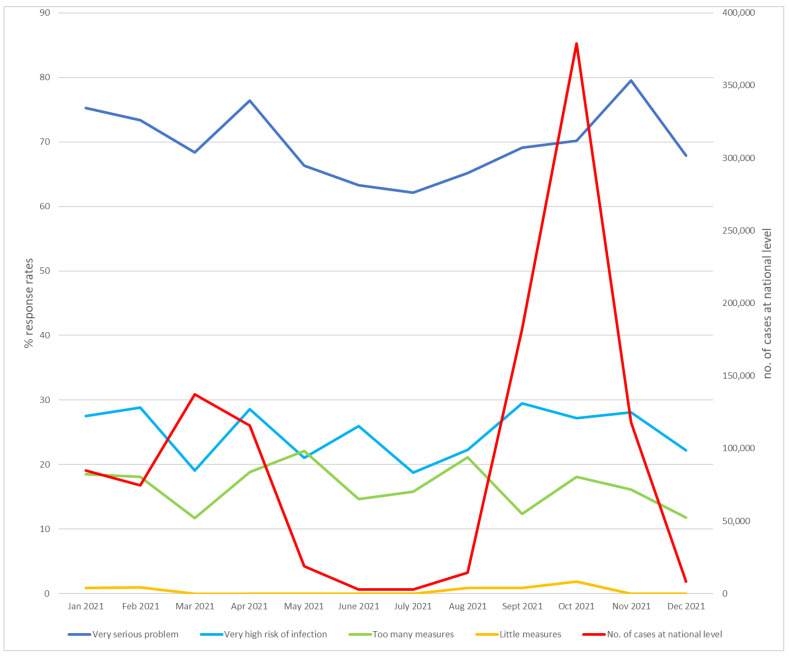
Evolution of the number of COVID-19 cases, infection risk perception, and perception of protective actions at the OCH in 2021.

In 2021, the perception of a “very serious problem” was still high, ranging between 58% and 79%, which is not different from 2020 (*p* = 0.5). The perception of a “very high risk of infection” decreased significantly to 24% in 2021 vs. 32% in 2020 (*p* = 0.01). An explanation of this could rely on the vaccination campaign that started in January 2021 (Figure 3).

**Figure 4:**
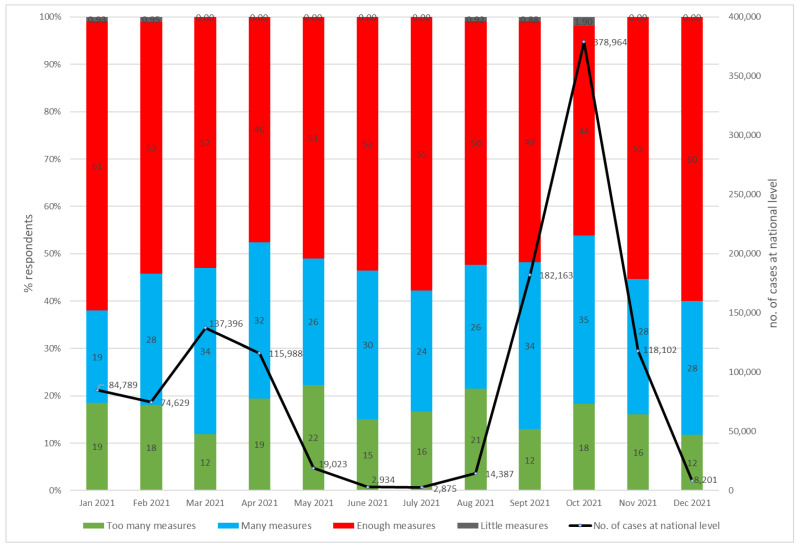
Opinions about protective actions at the OCH and the no. of cases in Romania in the period of January–December 2021.

The item “too many actions” did not increase significantly in 2021 compared with 2020 (*p* = 0.3). In 2021, the values ranged from 11% to 22% with an average of 16% compared with an average of 13% in 2020. The evolution of “too many actions” is inversely proportional to the number of cases, as we noted in 2020 (Figure 4). Toward the end of the year, the reported rates decreased incrementally to a value of 11%.

In 2021, the same pattern of responses for the open question was noticed. Averages were at 17% for Q4 and 18% for Q5 response ratios. Out of these responses, 87% stated that no other action should be taken (Q4) and the actions to implement were for Operational Personnel and System.

On question five (Q5), 73% of the respondents stated that no action should be dropped. From the affirmative recommendations, 4% to 8% were to drop mask, drop CT scan, or drop test, while Varia had 3%.

These findings provided the management with the confidence to keep in place the supplementary protective actions and the same standard operating procedures (SOPs) for PCR testing and lung CT scans before admission. The number of admissions increased in 2021 with 12% compared with 2020, and this proved that the overprotective strategy paid off, as is shown in Table 3.

Correlation analysis revealed population perceptions of the gravity of the problem and personal risk were similar in 2021 to 2020 findings. The “very serious problem” item directly and significantly correlates with the “very high risk of infection” (r^2^ = 0.689, *p* = 0.015) but not with the “number of cases” (r^2^ = 0.8364, *p* = 0.245). This was due to the influence of the vaccination against COVID-19 that started in January 2021 and less toward the Omicron (B. 1.1. 529) strain, which was first reported on 24 November 2021 [14].

## 4. Discussion

PREMs play a significant role in evaluating and influencing the success of a hospital or healthcare facility. A hospital’s success is not only determined by its clinical outcomes, but also by the overall patient experience. PREMs provide valuable information on how patients perceive the quality of care and services they receive, and this feedback can have a substantial impact on various aspects of hospital decisions and success.

But, in a crisis situation, such as the COVID-19 pandemic, the hospital had to adapt quickly, implement mandatory regulations, but also undertake every task possible to protect the patient. The patients, however, may have different opinions and perceptions; they may or may not have knowledge about medical subjects and are informed by the general media or social media [15]. This may cause differences between what the management believes and what the patient perceives [16]. This gap in knowledge must be reduced, and this can be performed through a continuous evaluation of PREMs collected from the hospital patients and through taking actions that are triggered by the findings.

In the pandemic period, patients’ perceptions of protective actions against COVID-19 varied greatly from “too many actions” to “little actions”. These perceptions are influenced by individual beliefs, experiences, and various factors [15]. In the situation of the COVID-19 pandemic, scientific evidence appeared over the course of weeks, if not even months. The time that passed between the appearance of scientific conferences, preprints, or scientific news and their implementation in practice was valuable time lost in the race against the virus [16]. This lag in information and evidence put an extra challenge on hospital management, since patient safety is one of the main concerns in hospitals all over the world. The same is also true for Romania, where there is a willingness to improve patient safety, especially if there are resources that can be invested in this direction [17]. The OCH management decision was to invest in resources and supplementary actions in order to increase patient safety during the COVID-19 pandemic, but this could come with the risk of losing patients (scared by too many protective actions). This type of decision is happening in many hospitals, and this is an example of how to manage such a situation with PREM utilization.

It has been shown that hospitals that take PREMs seriously and use feedback to improve the patient experience tend to achieve greater success [18]. This success includes not only financial performance but also a positive reputation, improved patient outcomes, and a more efficient and patient-centered system.

While PREMs are usually elaborate constructs, with many questions converging toward a common conclusion, there is also the situation when a one-question approach can give enough information for use and can even replace a multiple-item questionnaire [19]. In our case study, by using PREMs, we created a feedback mechanism that helped hospital management to successfully implement some very difficult and important actions in the hospital during a hard period of the COVID-19 pandemic.

## 5. Limitations and Strengths

This study does not attempt to exhaustively cover or explain all important factors that impacted patients’ experiences of COVID-19 protective actions at OCH. Factors such as those related to the hospital’s external environment including the impact of budget [20], the societal cost of disease [21], and complex factors that influence determinants of choice [22] or engagement in preventive behaviors [23] were beyond the purpose of this research. Developing PREMs is a complex and rigorous science and has clear guidelines [24,25,26]. Our study and documentation took all these guidelines into consideration during the development of the PREMs, but were limited by limited resources and the intense COVID-19 situation at the time. However, the rigorous process we followed has created PREMs that fit the purpose of informing management decisions and monitoring at the hospital and demonstrated the importance of using PREMs in crisis situations. Further research is needed to explore the hypothesis generated in this research to develop a more comprehensive tool that includes predictor factors for perceptions of medical care in planning for future health emergencies.

## 6. Conclusions

The patients’ appreciation of the COVID-19 protective actions taken by the hospital were related more to the general perceptions induced by the number of cases presented in the mass media and less by the perceptions of the gravity of the problem or the risk of infection. In a hospital, the primary mission of patient safety is essential, and it must be fulfilled even if the patients are not sure or fully convinced that this is for their benefit.

For management decisions and monitoring, using PREMs can be essential in a situation when general evidence is not conclusive. At the same time, it has been shown that using PREMs does not necessarily require sophisticated statistical tools and analyses.

## Figures and Tables

**Table 1:** Reclassification of the open question answers for Q4: What other actions to prevent the spread of COVID-19 do you think OCH could adopt? (If applicable).

	Reclassification	Free Text Open Question Answers
1.	None	*“None”, “not the case”, “Is OK”, “nothing more”, “no”*
2.	Operational	*“Too much paperwork’s”, “form three waiting lines”, “make better sharp scheduling”, “maximum 2 people with COVID-19 should be inside”, “make more single patient rooms”, “make a bigger waiting room”, “make a special looker room for patients”, “patient visit should be permitted to COVID-19 negatives one”, “install automatic shoes plastic protection device”*
3.	Personnel	*“Check if personnel respect rules”, “test periodically the personnel”, “more personnel at the triage”, “continuous personnel information and training”*
4.	Informational	*“More patients infographics”, “population is not correctly informed”,*
5.	System	*“change mentalities”, “stop parties, and irresponsible people gathering”, “local health authorities should be more involved”, “promote vaccination”*
6.	Varia	*“Is a virus that can’t be stopped”, “WHO, government and mass media are responsible for amplification of the danger”, “It is a total fraud on people money, working places should be developed”, “Services are too expensive”*

**Table 2:** Reclassification of the open question answers for Q5: In your opinion, which of the actions already adopted to prevent the spread of COVID-19 could OCH abandon (If applicable)?

	Reclassification	Free Text Open Question Answers
1.	None	*“none”, “negative”, “No”, “Not the case here”, “keep it as it is”*
2.	Masks	*“Mask wearing in the rooms”, “surgeons mask wearing during surgery, to not suffer for hypoxia”, “COVID-19 negatives patients to not wear masks”. “No mask”*
3.	CT scan	“*CT is useless irradiating for me*”, “*CT before admission should be dropped*”
4.	PCR	*“Not to be done twice per month to the same person”, “PCR test is expensive”*
5.	Triage	*“Waiting for triage outside the hospital”, “too much paperwork’s in the triage”, “Too much paperwork’s”, “allocate more personnel to the triage”*
6.	Varia	*“Chlorine solution usage”, “stop smoking”, “tougher actions are needed”, “cancel stop smoking actions”, “set up an all gathering relaxing place”, “this question is complicated”*

**Table 3:** Number of and year on year % increase in admissions.

Year	Admissions	% Year on Year Increase
2017	2207	
2018	2313	105%
2019	2379	103%
2020	2604	109%
2021	2906	112%

## Data Availability

Data available at https://osf.io/w9rnc (accessed on 10 November 2023).

## Appendix A. Questionnaire on Actions Applied by OCH to Protect AGAINST COVID-19

You do not have to sign, this questionnaire—is ANONYMOUS.

Complete the questionnaire by checking the box next to the chosen option.

Your opinion is very important.

The collected information helps us to increase the quality of the medical services offered and improve the patient experience at Ovidius Clinical Hospital

Please submit the completed questionnaire in the specially arranged place on the ward.

Your age _____

Sex □ male □ female

□ urban □ rural

Q1. What is your opinion regarding the current situation related to the corona virus infection (COVID-19)?

□ It’s a very serious problem, □ It’s a problem, but not that bad, □ It’s a small problem, □ It’s not a problem at all, □ No opinion

Q2. Are you afraid that you might get infected?

□ Very much, □ Much, □ A little, □ Not at all, □ No opinion

Q3. What do you think of the actions taken by OCH for protection against COVID-19?

Too many actions □ Many □ Enough □ Little □ No opinion □

Q4. What other actions to prevent the spread of COVID-19 do you think OCH could adopt? (If applicable)

Q5. In your opinion, which of the actions already adopted to prevent the spread of COVID-19 could OCH abandon (If applicable)?

Thank you for your time!
